# Supplementary material for: Compensatory growth and recovery of cartilage cytoarchitecture after transient cell death in fetal mouse limbs
Source: Nat Commun. 2024 Apr 5;15:2940. doi: 10.1038/s41467-024-47311-7 (PMC10997652; doi:10.1038/s41467-024-47311-7)

Compensatory growth and recovery of cartilage cytoarchitecture after transient cell death in fetal mouse limbs

Chee Ho H'ng, Shanika L. Amarasinghe, Boya Zhang, Hojin Chang, Xinli Qu, David R. Powell, Alberto Rosello-Diez.

SUPPLEMENTARY INFORMATION

Supplementary Data 1-4

These Tables present the Differential expression (DE) analysis for the RNA-seq experiment described in Supplementary Figure 7. Data from E15.5, E17.5, P0 and P3 are presented, respectively in Tables 1-4. Within each table, multiple comparisons are presented in different tabs. C, control; E_L, Exp Left, E_R, Exp Right.

Supplementary Figures and legends (start on next page)

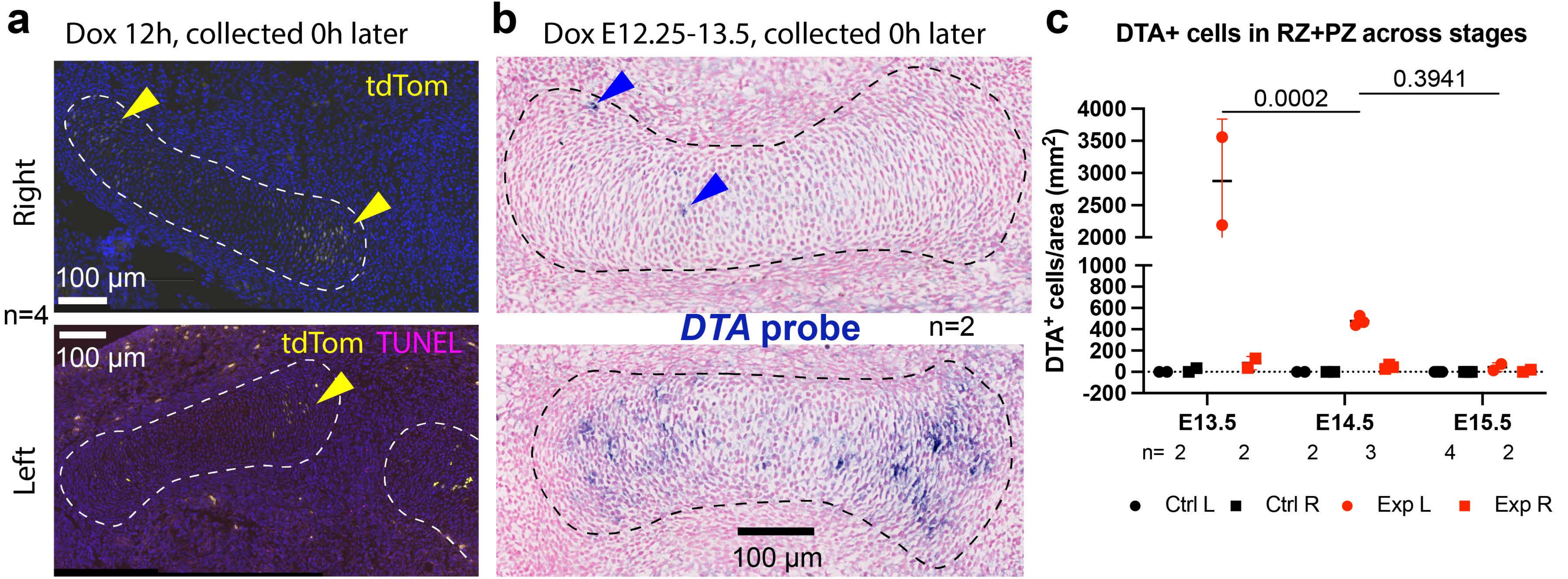

Supplementary Fig. 1. **a** Immunohistochemistry showing the expression of tdTomato (yellow arrowheads) achieved in the cartilage of the *Pit-Col-DTA* model (white dashed lines) after 12h Dox treatment. **b-c** *In situ* hybridisation showing the expression of *DTA* (blue arrowheads) achieved in the cartilage of the *Pit-Col-DTA* model (delimited by black dashed lines) after 30h (E13.5 time-point) or 36h (E14.5 & E15.5 time-points) of Dox treatment, and quantified in (**c**). Sample number (n) as indicated. p-values of Sidak's multiple comparisons test, after 2-way ANOVA.

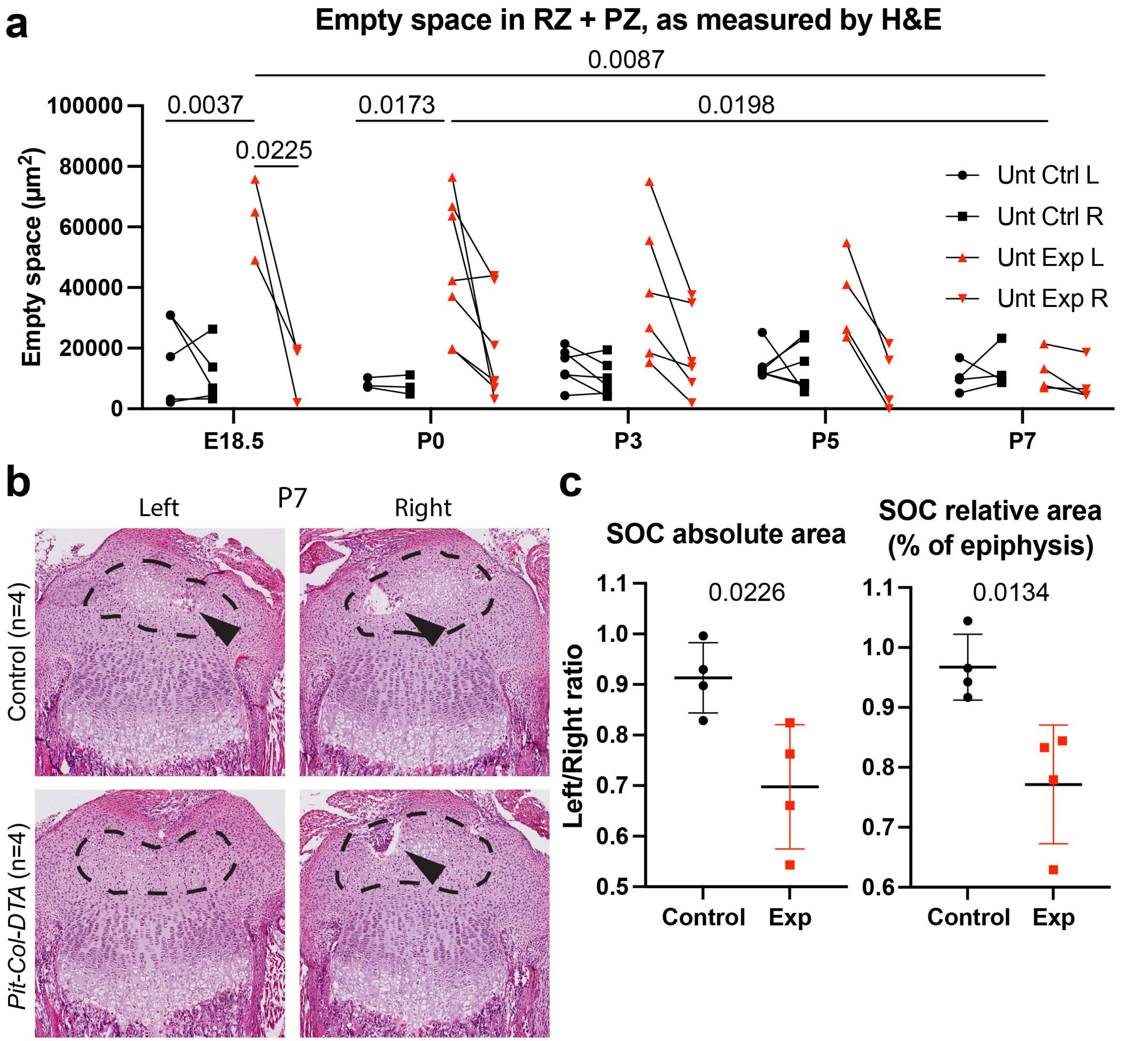

Supplementary Fig. 2. **a** Quantification of empty space area in the resting + proliferative zone (RZ + PZ) of left and right proximal tibia in Ctl and Exp samples, at the indicated stages. 2-way ANOVA for Genotype and Side followed by post-hoc multiple-comparisons test. p-values shown in the graph. **b** H&E staining on sections of proximal tibia at P7 showing the formation of the secondary ossification centre (SOC, dashed lines) and the invasion of blood vessels (arrows). **c** Quantification of the left/right ratio of the absolute area (µm^2) of the SOC and blood vessel invaded region (left) and the relative area of SOC plus invaded region over the epiphysis area, at P7. p-values for unpaired t-tests are shown, n=4 Ctl and 4 Exp.

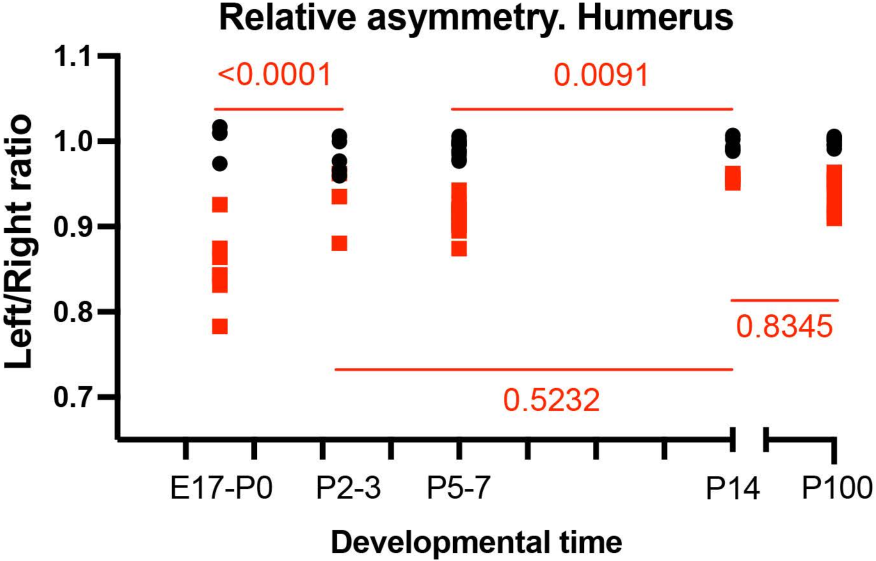

Source of Variation	% of variation	P value
Interaction	10.64	<0.0001
Stage	8.574	<0.0001
Genotype	47.35	<0.0001

**Control** vs **Exp**	
E17-P0	<0.0001
P2-3	0.0046
P5-7	<0.0001
P14	0.0406
P100	<0.0001

Supplementary Fig. 3. Left/right ratio of the humeri length for control (black) and *Pit-Col-DTA* specimens (red) at E17-P0 (n=4 Ctl, 7 Exp), P2-P3 (n=5,3), P5-P7 (n=11,9), P14 (n=5,4), P100 (n=8,7). Values for 2-way ANOVA are shown in the Table on the top right. p-values of Sidak's multiple comparisons tests are shown on the graph and the bottom right table.

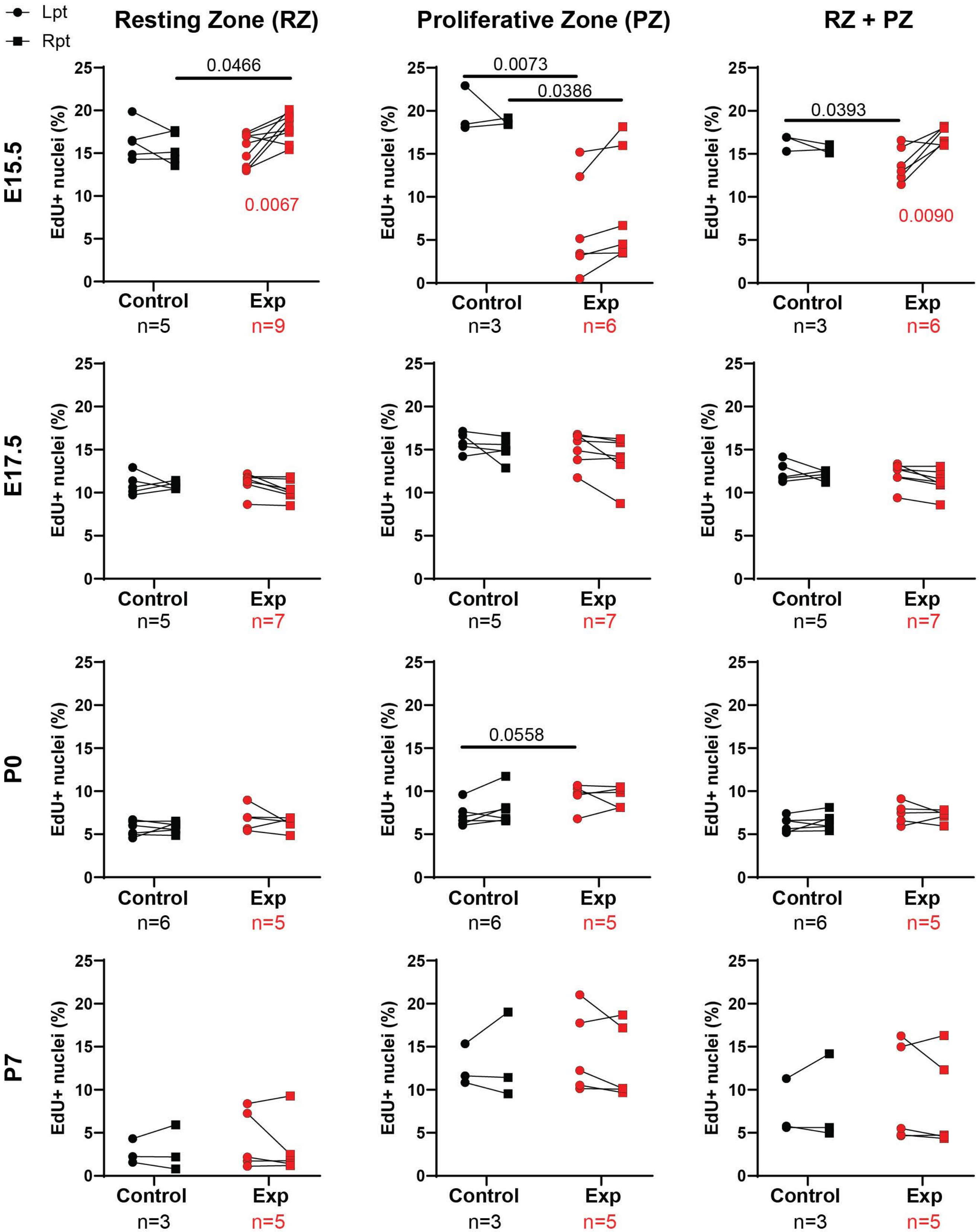

Supplementary Fig. 4. Graphs showing the percentage of EdU⁺ nuclei/total nuclei of either the RZ, the PZ or both combined, at the indicated stages. Statistical analyses are 2-way ANOVAs and Sidak's post-hoc multiple comparisons tests, performed at each stage for all zones, in a pairwise manner. Sample number (n) as indicated.

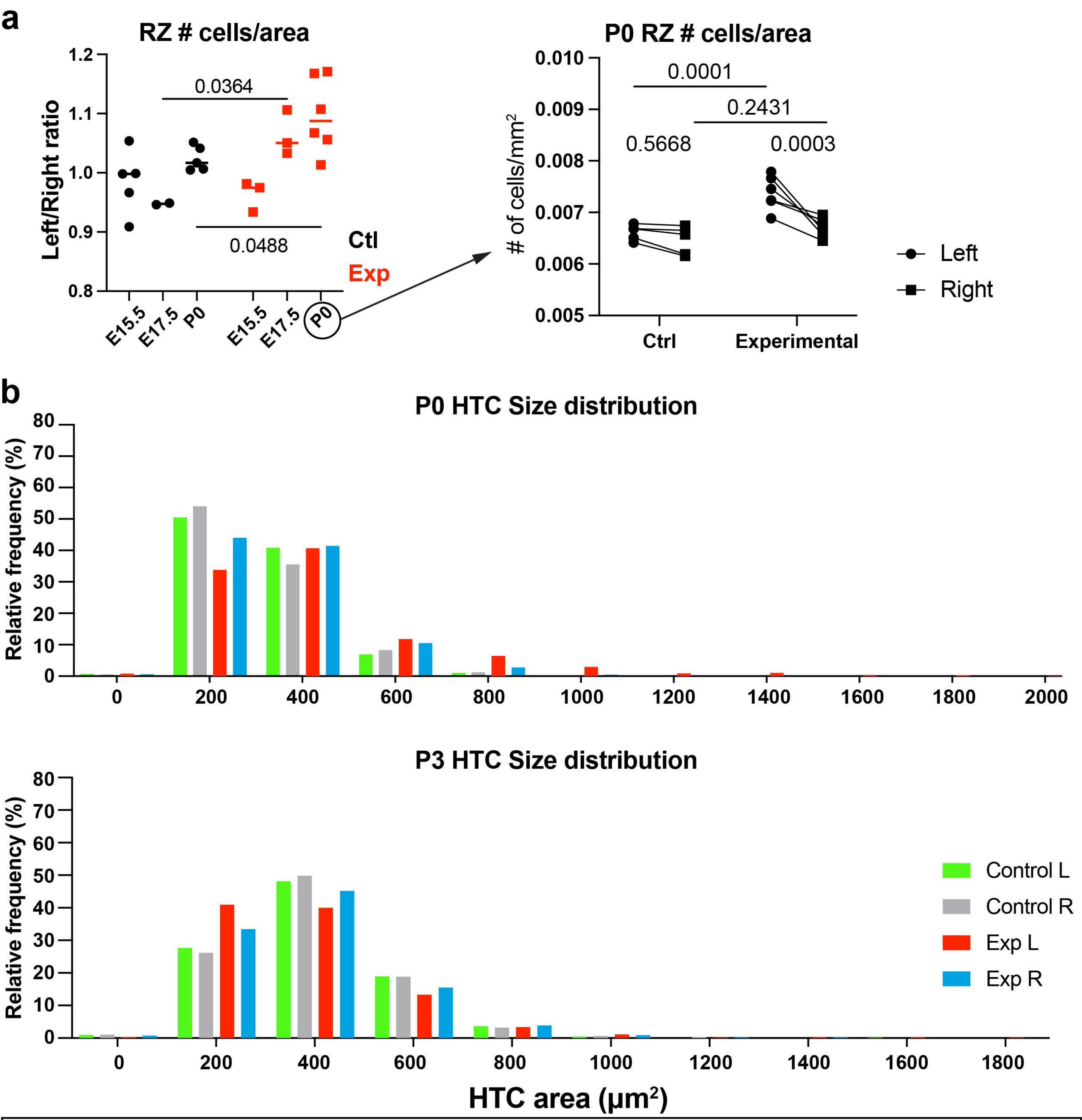

Supplementary Fig. 5. **a Left**, Graphs showing the left/right ratio of the number of cells in the resting zone per area unit at E15.5 (n=5 Ctl, 3 Exp), E17.5 (n=5,3), P0 (n=5,6). Right, Graph showing the number of cells in the resting zone per area unit at P0. **b** Histogram of the cell size frequency distribution of the hypertrophic chondrocytes for Ctl and *Pit-Col-DTA* specimens at P0 (n=3 Ctl, 3 Exp), P3 (n=3,3).

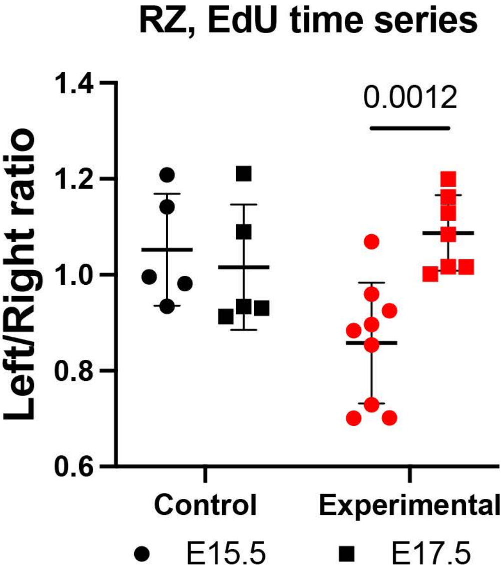

Supplementary Fig. 6. Graphs showing the left/right ratio of the instantaneous EdU incorporation in the resting zone (RZ) chondrocytes for Ctl and *Pit-Col-DTA* mice at E15.5 (n=5 Ctl, 9 Exp) and E17.5 (n=5, 7). Statistical analyses are 2-way ANOVA and p-values of Sidak's multiple comparisons tests shown on the graph.

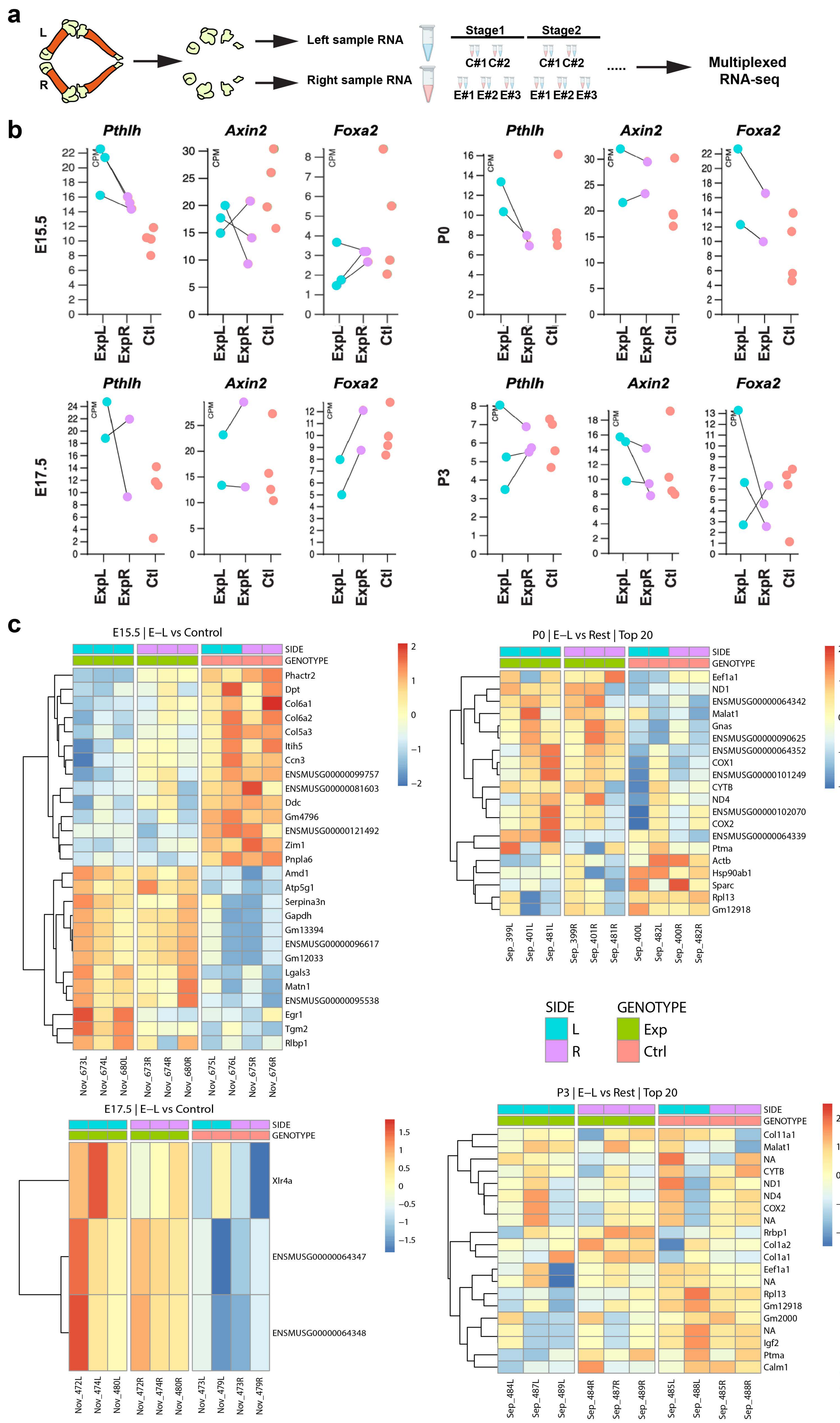

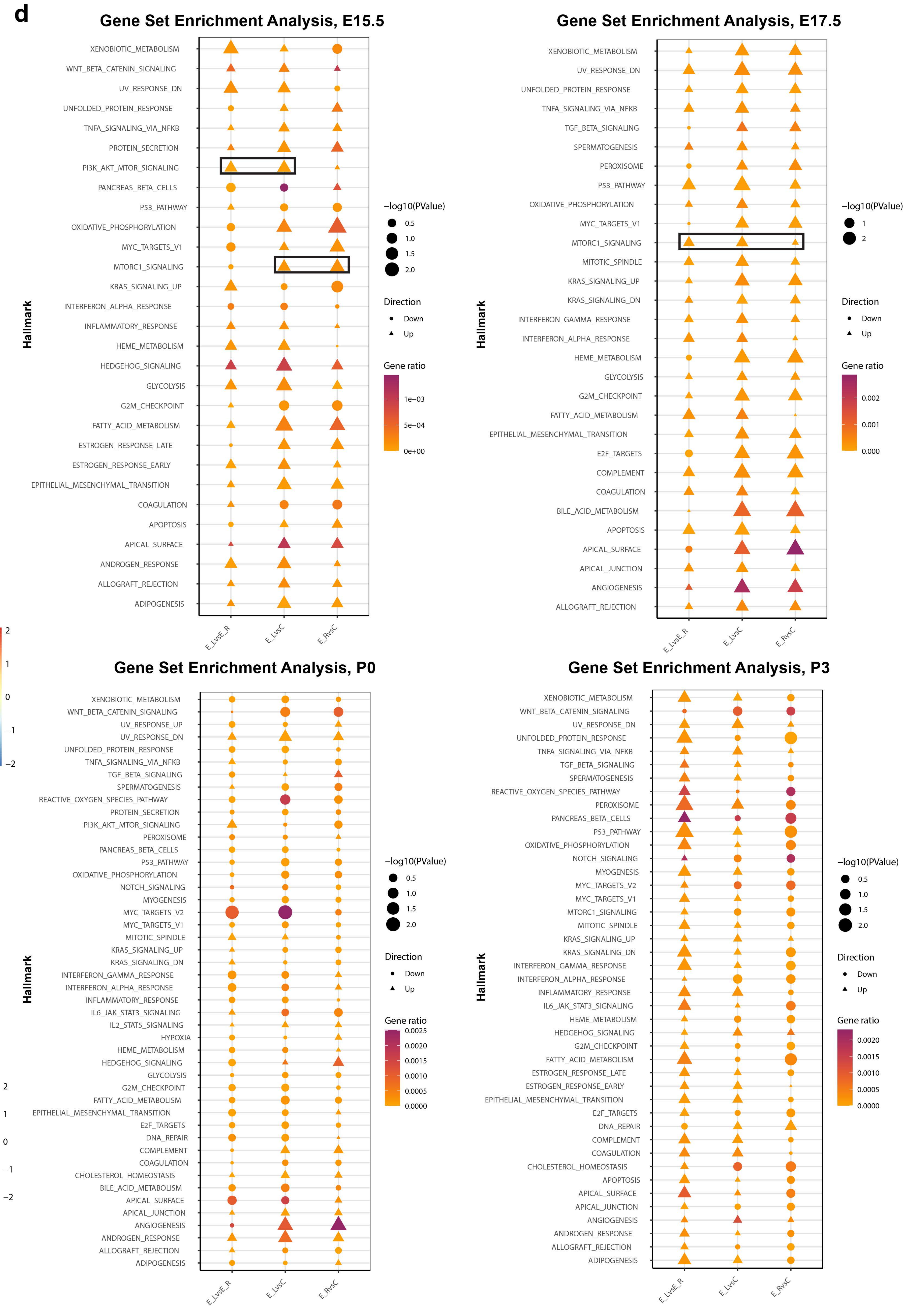

Supplementary Fig 7. a Schematic showing the cartilage region collected and processed for the bulk RNA-sequencing analysis. **b** Expression of *Pthlh, Axin2* and *Foxa2*, presented as counts per million (CPM) at E15.5 (n=2 Ctl, 3 Exp), E17.5 (n=2,3), P0 (n=2,3) and P3 (n=2,3), analysed using Degust software. **c, d** Heatmaps of the Differential Gene Expression (DGE, c) and Gene Set Enrichment Analyses (GSEA, d) at indicated stages as described in the methods.

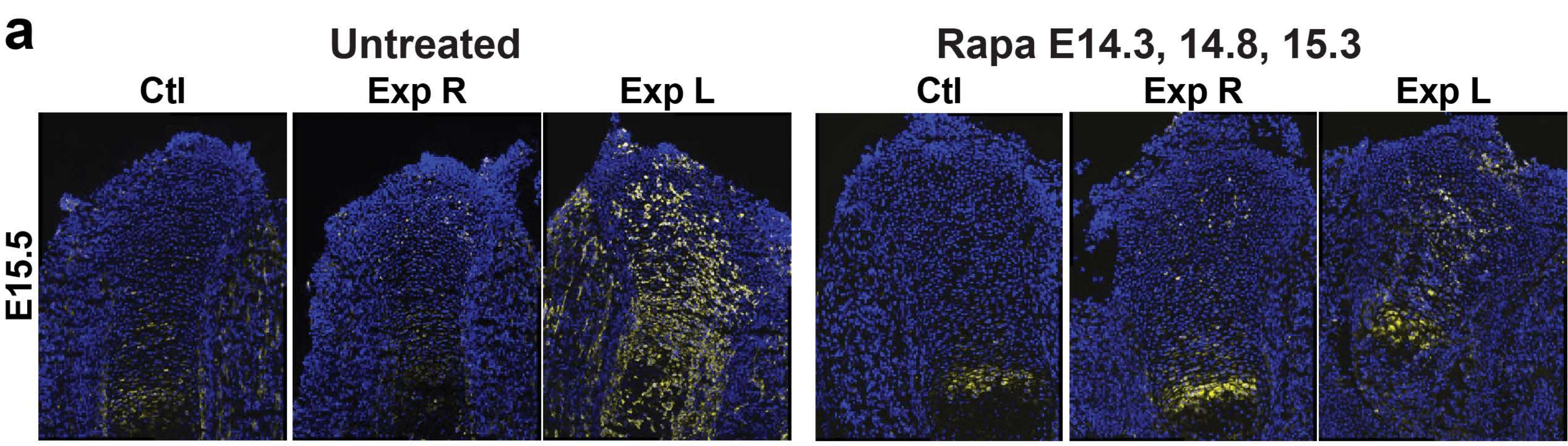

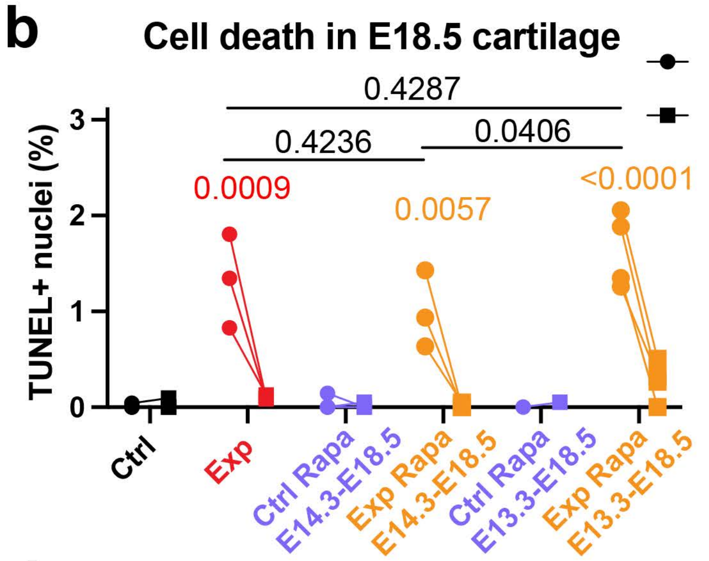

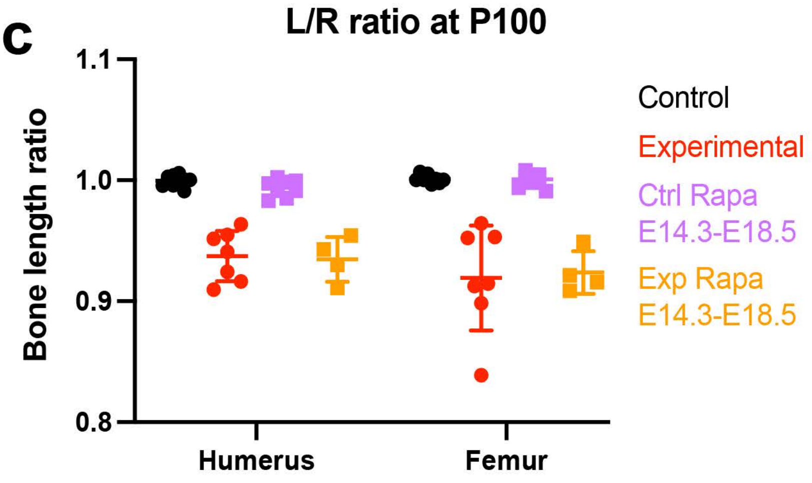

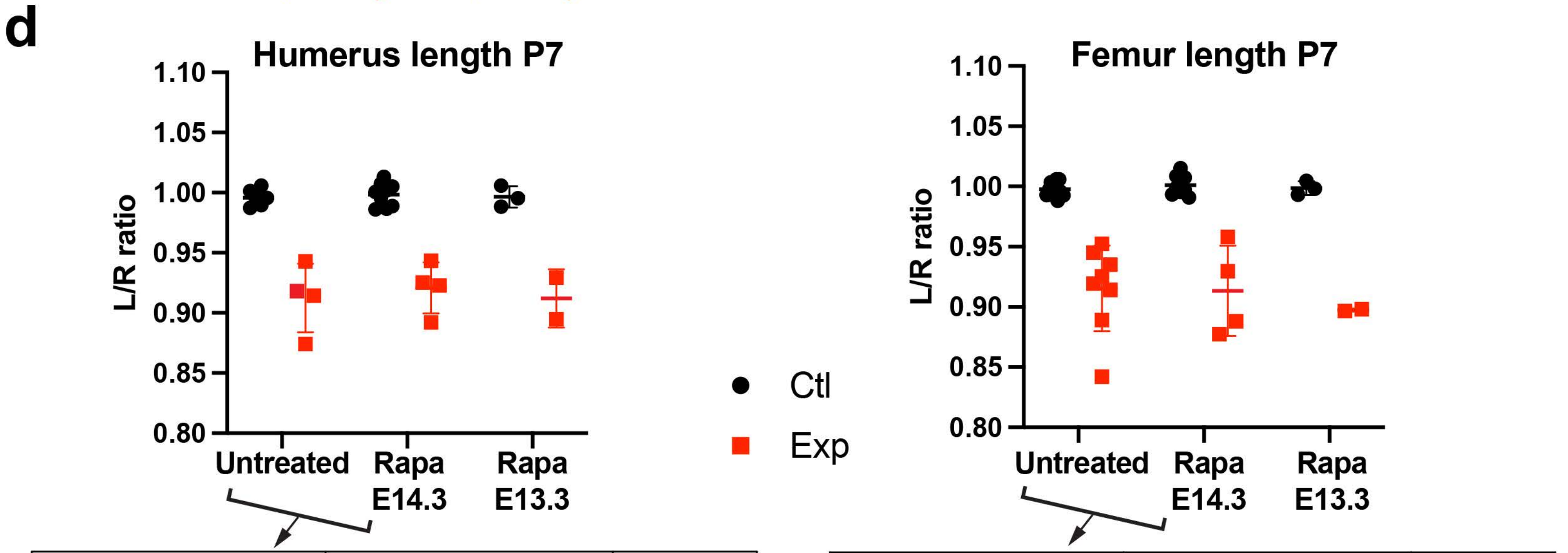

Source of Variation	% of total variation	P value
Interaction	0.1240	0.6657
Genotype	85.59	<0.0001
Treatment	0.3911	0.4453

Source of Variation	% of total variation	P value
Interaction	0.07676	0.7488
Genotype	74.21	<0.0001
Treatment	0.005904	0.9292

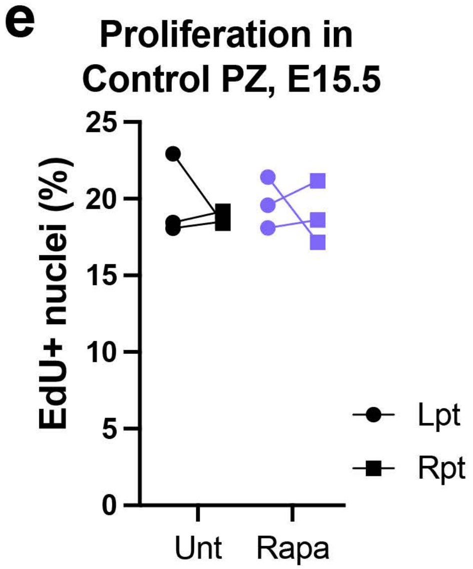

Supplementary Fig. 8. a Immunohistochemistry for p-S6 (yellow) in Untreated (n=5 Ctl, 6 *Pit-Col-DTA*) and Rapamycin-treated (Rapa, n=4,6), specimens at E15.5. **b** Quantification of TUNEL+DAPI+ cells at the indicated stage and genotypes, in control or Rapa-treated litters. p-values for posthoc multiple comparisons tests after 2-way ANOVA are shown. **c** Graphs showing the left/right ratio of the length of P100 humerus (Untreated, n=8,7; Rapa, n=8,4) and femur (Untreated, n=8,7; Rapa, n=8,4) as indicated. Values for 2-way ANOVA are shown in the Table on the bottom of each graph. **d** Like (c),but for P7 (humerus: Untreated, n=6,4; Rapa, n=9,4; femur: Untreated, n=9,8; Rapa, n=9,4). No significant differences found. **e** EdU+ nuclei in the E15.5 PZ of Control animals, either untretaed or Rapa-treated (n=3 each). EdU was injected 1.5h before tissue collection.

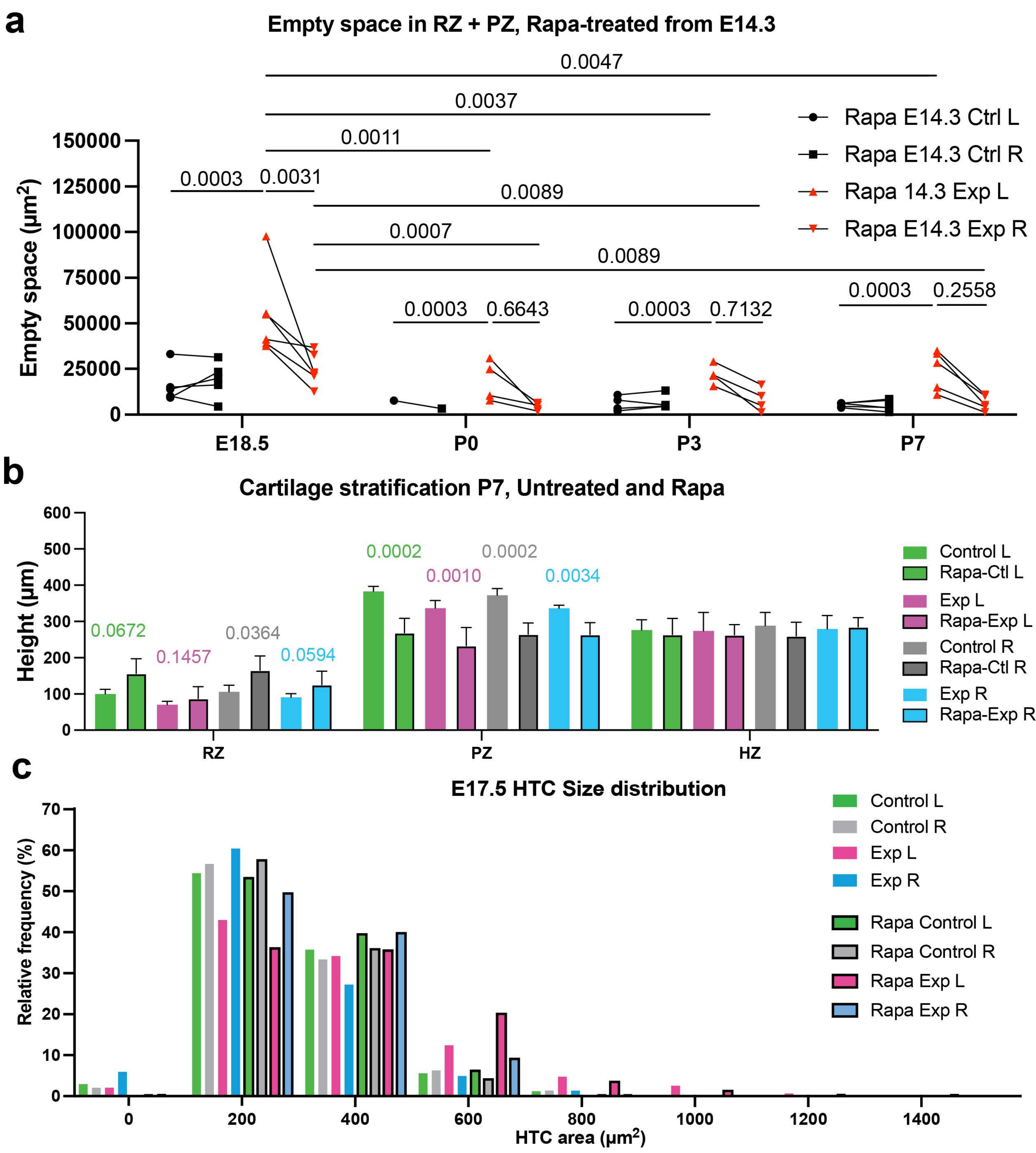

Supplementary Fig. 9. **a** Quantification of empty space area in the resting + proliferative zone (RZ + PZ) of left and right proximal tibia in Ctl and Exp samples, in Rapa-treated animals at the indicated stages. 2-way ANOVA for Genotype and Side followed by post-hoc multiple-comparisons test. p-values shown in the graph. **b** Absolute length (µm) of the different cartilage zones (proximal tibia) in P7 animals from untreated litters or treated in utero with Rapa. p-values for the multiple comparisons tests (after pairwise 2-way ANOVAs) are shown. **c** Distribution of HTC sizes in the proximal tibia, for the indicated genotypes, treatments and sides, at E17.5 (n=3 Ctl and 3 Exp Untreated samples, 3 Ctl and 5 Exp Rapa-treated samples). Genotypes and sides are colour-coded.

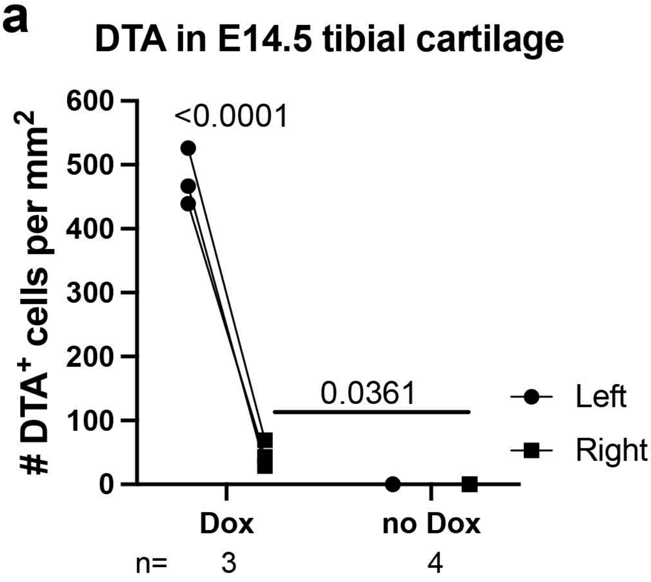

Supplementary Fig. 10. a, Number of DTA$^+$ cells per area, identified in the E14.5 left and right cartilage of *Pit-Col-DTA* animals, either treated (n=3) or not (n=4) with Dox. **b-b’’’**, p-S6 immunostaining in left and right ribs of Control and Exp animals (n=2 embryos each, 3 ribs per side). Dashed lines delimit the cartilage. Boxed regions are shown magnified 2.5x below (b’, b’’, b’’’) including TUNEL signal (absent in the cartilage). p-values for Sidak’s multiple comparisons test (after 2-way ANOVA) are shown.

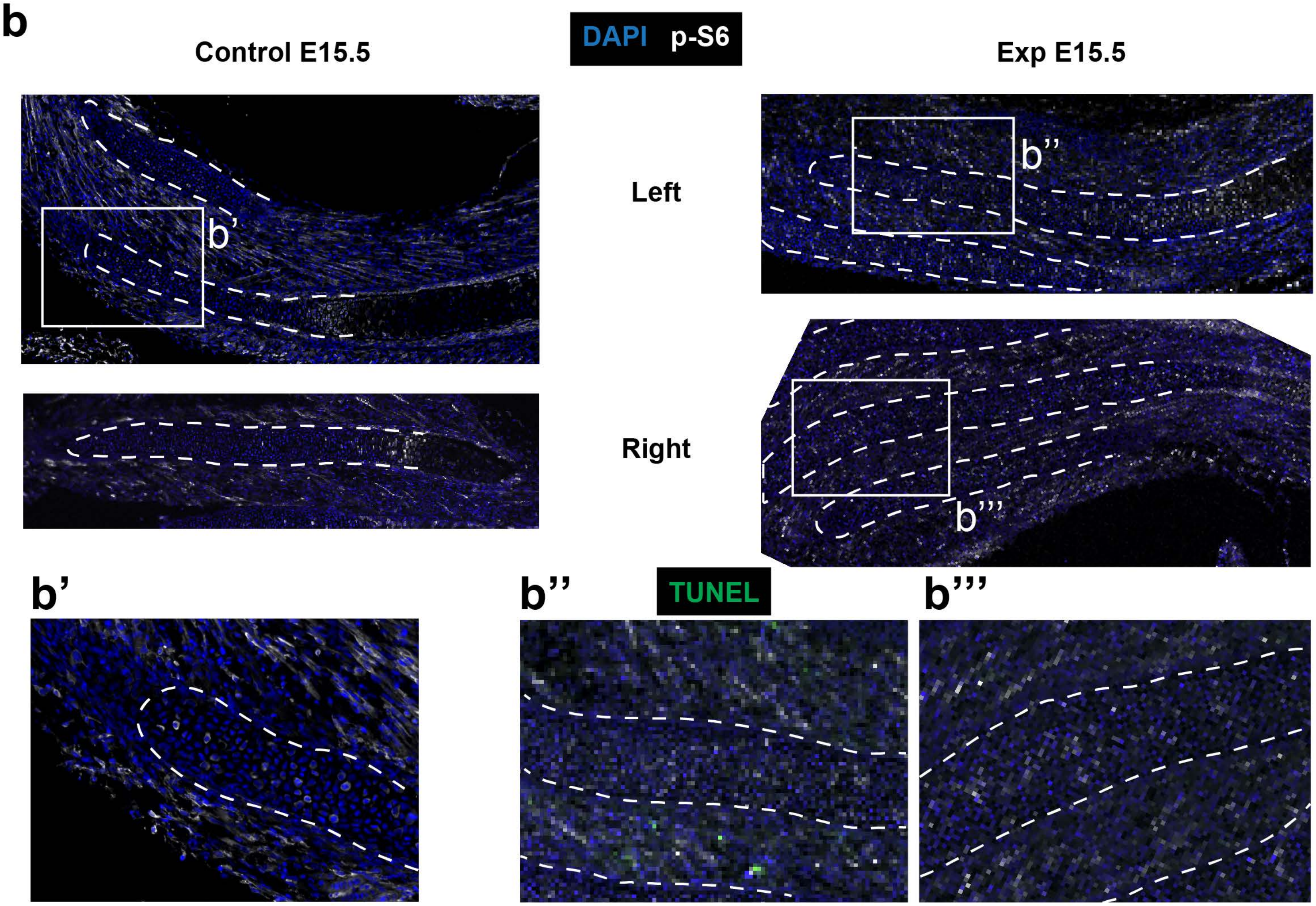

Supplement: Supplementary file 1 — Supplementary Information [file 41467_2024_47311_MOESM1_ESM.pdf]
